# The global rise in Parkinson's disease: a critical analysis of causes and future directions

**DOI:** 10.3389/fpubh.2025.1606732

**Published:** 2025-06-04

**Authors:** Jose E. Leon-Rojas

**Affiliations:** Grupo Cerebro, Emoción y Conducta, Escuela de Medicina, Universidad de las Américas (UDLA), Quito, Ecuador

**Keywords:** Parkinson's disease, toxins, environmental toxic pollutants, pesticide and insecticide, exposome

## Introduction

**Figure 1:**
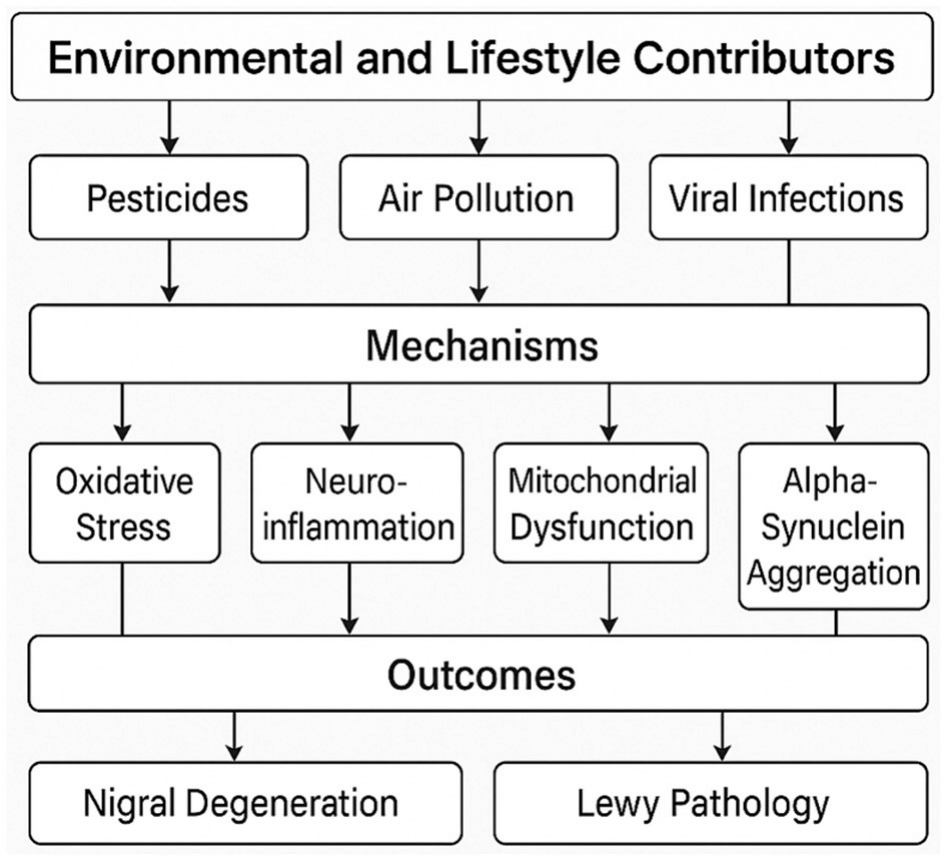
A conceptual framework of environmental and lifestyle contributors to the rising PD incidence.

An important challenge in neurology today is the worldwide dramatic rise in Parkinson's disease (PD) incidence. Epidemic studies over the past few decades have revealed a concerning trend: PD is not only becoming more common due to aging populations but also increasing in age-adjusted analyses, implying that demographic changes by themselves cannot explain this surge (1, 2). This trend is both alarming and intellectually provocative—why is a disease first reported over two centuries ago now accelerating in frequency? The solution, I contend, is a complicated interaction of environmental, genetic, and lifestyle elements many of which have been magnified by modern industrialization. This paper calls for a paradigm change in how we approach PD prevention and research, critically reviews the data behind these possible causes, and identifies gaps in our understanding. I have provided a conceptual framework of the main environmental and lifestyle contributors to the rising incidence of PD in Figure 1.

## The aging population: an unfinished story

Unquestionably, aging is the main risk factor for PD; incidence rates sharply rise after the age of 60 (3). Particularly in high- and middle-income countries, the absolute number of PD cases will surely rise as world life expectancy rises. Still, this demographic justification falls short. In 2016, the global prevalence of PD was 6.1 million [95% uncertainty interval (UI), 5.0–7.3] (1) which has increased to 11.8 million (95% UI, 10.4–13.4) according to the 2021 global, regional, and national burden of disease study (2, 4). This increase was not solely due to an increase of the aging population, certainly age-standardized prevalence rates (per 1,000,000) show a stark picture, with an increase of 21.7% (95% UI, 18.1–25.3) between 1990–2016 and of 16.1% (95% UI, 13.8–18.5) between 2010–2021 (1, 2, 4). These studies, adjusting for age, show that PD cases are rising even in younger populations, suggesting that other, maybe modifiable elements are in play. We would expect rates to stabilize in nations with plateauing life expectancy if aging were the only factor; yet, this is not the case (5, 6). This discrepancy implies that other factors are actively increasing the risk even if aging prepares the ground for neurodegeneration.

## Environmental toxins: the silent accelerants

Among the most convincing—and unsettling—justifications for the increase in PD is the explosion of environmental neurotoxins. Supported by both epidemiological and mechanistic studies, the link between pesticides including paraquat and rotenone and PD risk is among the best-documented in the field (7–9). Although it is banned in the EU and China, paraquat, an herbicide still used in many nations, has been repeatedly linked to PD in epidemiological research. According to a 2011 case-control study, those with PD had a positive association with paraquat exposure with an OR of 2.5 (95% CI, 1.4–4.7); the association rises even more in those with particular genetic susceptibilities (7). Another pesticide, rotenone, directly reduces the function of the mitochondrial complex I in ways that eerily match the biochemical dysfunction of PD (9). Exposing animal models to rotenone results in Lewy body-like inclusions and nigrostriatal degeneration, offering a direct experimental link between toxin exposure and PD pathology (8, 9). Still, regulatory responses have been utterly inadequate. Even as peer-reviewed studies mount, the Environmental Protection Agency (EPA) in the United States has regularly delayed action on paraquat. With the strongest links for paraquat, rotenone, and organochlorines such as dieldrin, a 2018 meta-analysis found that occupational exposure to pesticides raises PD risk by at least 5%−11% at 5 and 10 years of pesticide exposure, respectively (10). The latency between exposure and symptom onset complicates public awareness and lets these toxins stay in use while their neurological effects go unseen. Regulatory authorities have been slow to respond, sometimes citing inadequate “proof” of causality—a criterion almost impossible to satisfy in chronic, multifactorial diseases.

Once everywhere in dry cleaning, adhesives, and metal degreasing, trichloroethylene (TCE) has become another likely offender. Individuals with occupational TCE exposure had a six-fold increased risk of acquiring the disease, according a 2011 study of twins discordant for PD (11). More concerning still, TCE persists in soil and groundwater for years, so communities close to former industrial sites could be continuously exposed. Related solvent perchloroethylene (PCE) has also been linked to PD; evidence points to even low-level exposures over time possibly being sufficient to cause neurodegeneration (12). These compounds have long been used in industrial environments, which has produced a quiet reservoir of risk. For decades, TCE and PCE contaminated Camp Lejeune, a U.S. Marine Corps base, and later a ground-breaking study, published in 2023, found higher rates of PD among veterans housed there and reported a staggering 70% increase in the risk of PD for those exposed (13). These cases highlight the long-term effects of environmental neglect—exposures from the 1970s are only now showing up as illness. Certainly, research is complicated by the latency between exposure and symptom onset, but it also means we might be seeing only the start of a wave of poison-driven PD cases.

The impact of air pollution on PD is a newer but rapidly growing area of concern. Increased PD incidence in several epidemiological studies has been linked to fine particulate matter (PM2.5) and traffic-related pollutants. People living in areas with high PM2.5 levels had a 9% higher risk of PD compared to those in cleaner areas, according to a 2016 study in Denmark (14). Experimental studies point to airborne toxins causing systemic oxidative stress, promoting neuroinflammation and alpha-synuclein aggregation (15). Urbanism and industrialization are aggravating this issue. In fast developing nations like India and China, where air quality is often seriously compromised, PD incidence is rising in line with pollution levels (1, 16). Air pollution is an unavoidable exposure for billions of people unlike pesticides or solvents, making it a particularly insidious public health challenge.

## Genetic susceptibility: a little actor in a big epidemic

Although genetic studies have found important mutations—e.g., in LRRK2, GBA, and SNCA that increase PD risk—these represent only a tiny portion of cases (17). The primacy of environmental and lifestyle factors is reinforced by the clear rise in PD incidence over just a few generations far exceeding any possible genetic drift. Said another way, gene-environment interactions remain a vital frontier. For instance, carriers of GBA mutations could be particularly sensitive to pesticide exposure, yet most research misses these complex interactions (18). Although monogenic forms of PD (e.g., SNCA, LRRK2, PRKN mutations) represent a minority of cases, polygenic risk is increasingly recognized as an important contributor. Recent GWAS have identified over 90 risk loci, suggesting a complex polygenic architecture involving lysosomal function, mitochondrial maintenance, and autophagy pathways (17, 19). Polygenic risk scores (PRS), aggregating common variants, now allow stratification of individuals at elevated lifetime risk, with predictive potential enhanced when combined with environmental exposure data (19). For example, LRRK2 G2019S carriers may show increased vulnerability to pesticide exposure, supporting a gene-environment synergy (20). Additionally, sex-specific genetic architectures are emerging, with differing heritability estimates and variant penetrance by gender (21). These advances argue for more integrative risk modeling, embracing both biological inheritance and environmental exposure. However, the overreliance on wide-ranging genome-wide association studies (GWAS) has, in my opinion, eclipsed the need of more complex models combining environmental exposure data with genetic risk profiling.

## Factors of lifestyle: protection or illusion?

Among the most confusing results in PD epidemiology are the apparently protective effects of caffeine intake and smoking (22). Although these links have been repeated in several studies, suggesting smoking as a preventive tool is ethically and medically unacceptable. Whether nicotine's neuroprotective qualities or caffeine's modulating of adenosine receptors, the processes behind these connections remain hypothetical and not applicable in an ethical and widespread public health strategy against PD.

The data on diet and PD remain murky. While Mediterranean diets correlate with lower risk in observational studies (23), randomized trials are lacking. Urate, a potent antioxidant found in coffee and certain foods (e.g., organ meats, seafood), shows promise; higher serum and cerebrospinal fluid urate predicts slower PD progression in men (24). Yet urate-elevating therapies failed in clinical trials, illustrating the pitfalls of extrapolating from biomarkers to interventions (25).

Physical activity's protective role is less controversial, with meta-analyses linking regular exercise lower PD risk with a relative risk of 0.77 (95% CI, 0.70–0.8) (26). Exercise has no known negative effects, unlike caffeine or smoking; it also has pleiotropic effects for metabolic and cardiovascular health. Animal models show exercise increases brain-derived neurotrophic factor (BDNF) and mitochondrial biogenesis in the substantia nigra, so offsetting PD pathology (27, 28). Particularly striking is the dose-response relationship: a 2018 meta-analysis showed that for each 10 metabolic equivalent of task-hours/week increase of vigorous exercise the risk of PD decreased by 17% in men; such association was not found in women (29). For those with formerly inactive lifestyles, even starting midlife exercise seems to help (30). The reported sex discrepancy in the protective effects of physical activity, beneficial in men but not statistically significant in women, may arise from multiple interacting factors. Hormonal differences, particularly oestrogen's neuroprotective effects, may buffer PD risk in women independently of physical activity (31). Furthermore, differential reporting accuracy and activity intensity between sexes might bias results (32). Finally, neuroimaging studies suggest sex-based differences in dopaminergic system plasticity, which could modulate responsiveness to exercise (33). Clarifying these pathways is essential for tailoring preventive strategies in both sexes.

## The gut-brain axis: a new frontier or a red herring?

Based on results of alpha-synuclein pathology in the enteric nervous system and changes in gut microbiota composition in PD patients, the theory that PD may start in the gut has acquired popularity (34). Though mostly hypothetical, the so-called “dual-hit” theory suggests that a pathogen or toxin passes via the vagus nerve into the brain (35). Although gut dysbiosis has been shown in animal studies to affect neuroinflammation, we still lack clear evidence that the gut microbiome starts PD in humans. With some researchers advocating early probiotics or fecal transplants as treatments, the current explosion of interest in this field runs the danger of outpacing the data. We desperately need thorough longitudinal studies, tracking the changes in microbiome years before PD starts in order to provide useful information regarding any potential links with its pathogenesis or future avenues of treatment.

## Viral infections: an overlooked trigger?

The COVID-19 epidemic has sparked once more interest in viral causes of neurodegeneration. Historical reports of post-encephalitic parkinsonism following the 1,918 influenza epidemic point to viruses as indeed able to induce parkinsonian syndromes (36, 37). Available evidence revives these concerns; for instance, a 2015 study that included 131 PD participants found that IgG seropositivity to HSV-1 (*p* = 0.046), was significantly associated with PD after adjusting for age, gender, and education (38). In this study, logistic regression analysis showed that PD participants had a higher infectious burden (with both virus and bacteria) than controls with an OR of 1.86 (95% CI, 1.38–2.52) and an OR of 1.628 (95% CI, 1.05–2.53) for viral burden only (38). Similarly, infection with West Nile virus, influenza A, and herpesviruses has been associated with transient or permanent parkinsonian features (39, 40). SARS-CoV-2′s neuroinvasive potential, via the olfactory nerve or blood-brain barrier, can induce sustained neuroinflammation, oxidative stress, and α-synuclein upregulation (36, 41). In murine models, post-COVID brains show microglial activation and dopaminergic neuronal loss reminiscent of prodromal PD (36). Though longitudinal human data are scarce, a huge prospective follow-up of 236,379 COVID-19 survivors showed increased incidence of new-onset parkinsonism within 24 months, with an incidence of 0.11% (95% CI, 0.08–0.14) for the whole cohort and an incidence of 0.26% (95% CI, 0.15–0.45) for those admitted to the intensive care unit (42). These findings underscore the urgency of including viral history in PD risk modeling and biomarker research. Certainly, the more general question is if by priming neuroinflammatory pathways, viral infections could be silent contributors to PD risk. This field has not received enough attention partly because it is difficult to link past infections to later neurodegeneration.

## Important knowledge gaps and a call to action

There are several obvious gaps in PD research at present. First, most environmental risk studies rely on retrospective designs (case-control), which are vulnerable to recall bias. An essential but logistically difficult solution could be prospective exposome studies, combining geospatial toxin data with biomarker analysis. Second, partly because geneticists and environmental epidemiologists sometimes operate in research silos, gene-environment interactions remain understudied. Third, although mechanistic studies in animal models have clarified possible pathways, their applicability to human PD is usually overstated.

I argue going forward for three fundamental changes in research priorities:

1. Policy-driven prevention: regulatory authorities have to apply a precautionary principle, limiting or outlawing high-risk chemicals even before absolute proof is found; even more so given the existence of significant evidence linking pesticides and industrial solvents to PD.
2. Incentivize longitudinal human studies: establishing causality requires extensive cohorts that can properly track environmental exposures, microbiome changes, and viral infections over decades. These types of studies should be incentivized by relevant academic agencies and scientific stakeholders.
3. Interdisciplinary collaboration: developing a unified model of PD pathogenesis will depend on breaking down boundaries between geneticists, environmental scientists, and clinicians to foster impactful interdisciplinary work.

## Conclusion

Parkinson's disease's increasing prevalence is not an inevitable result of aging but rather a preventable crisis driven by modern living, industrialization, and environmental damage. Although studies have pointed up important risk factors, the field has been slow to apply these results into practical guidelines due to the lack of attention to this pressing issue. Scientists have to go beyond just recording correlations and, instead, support preventive actions and multidisciplinary work. Passive observation is over; the PD epidemic calls for an active response.
